# Six‐Minute Walk Test Is Superior to Grip Strength as a Marker of Functional Recovery During Cancer Cachexia Rehabilitation

**DOI:** 10.1002/jcsm.70024

**Published:** 2025-07-29

**Authors:** Addison Barber, Amber Willbanks, Kathryn Abplanalp, Christopher W. Lewis, Ben Binder‐Markey, Prakash Jayabalan, Richard L. Lieber, Ishan Roy

**Affiliations:** ^1^ Shirley Ryan AbilityLab (formerly known as Rehabilitation Institute of Chicago) Chicago Illinois USA; ^2^ Hines VA Medical Center Maywood Illinois USA; ^3^ Department of Physical Therapy and Rehabilitation Sciences Drexel University Philadelphia Pennsylvania USA; ^4^ Department of Physical Medicine and Rehabilitation Northwestern University Chicago Illinois USA; ^5^ Robert H. Lurie Cancer Center Northwestern University Chicago Illinois USA

**Keywords:** cachexia, grip strength, physical function, six‐minute walk test

## Abstract

**Background:**

Decline in functional independence is a defining event of cancer cachexia, and attempts at creating cachexia‐specific therapies have largely failed because of the inability to identify treatments that improve functional capacity. This may be, in part, due to a lack of outcomes that are appropriate and sensitive enough to detect functional recovery. Grip strength is a frequently used outcome measure in cachexia clinical studies; however, the use of gait‐based measures is now emerging. These two outcome measures have never been directly compared in the same cohort of cachexia patients regarding their ability to measure and relationship to functional independence. We hypothesize that gait‐based measures more comprehensively act as a proxy measure for functional independence related to cachexia.

**Methods:**

In a retrospective cohort study of 485 cancer patients with a range of cachexia severity and related functional decline who required care at a single‐centre inpatient rehabilitation facility (IRF), we assessed the six‐minute walk test (6MWT) and hand grip strength (hGS) as proxy measures for functional capacity. Functional capacity is defined as mobility and activities of daily living (ADLs), is quantified by measures of functional independence and referred to here as the Total Motor Score. Cachexia patients were identified primarily using the Fearon et al. consensus criteria, with secondary identification by the Weight Loss Grading Scale (WLGS), Prognostic Nutritional Index (PNI) and neutrophil‐to‐lymphocyte ratio (NLR). Primary outcomes were change/gain in Total Motor Score, IRF discharge destination (e.g., homebound status or need for care facility) and 6‐month survival.

**Results:**

The presence of cachexia in this cohort was 63%. This cohort was 52% male. Mean age was 63 ± 0.63 (SEM) years. Multivariate linear regression demonstrated that change in 6MWT (*p* < 0.0001) but not hGS (*p* = 0.084) correlated with Total Motor Score gain after controlling for age, disease burden, cancer type, previous cancer treatment and baseline motor function as covariates. Area under the curve analysis revealed that change in 6MWT (*p* < 0.0001, AUC = 0.77) was a stronger predictor of Total Motor Score gain than hGS (*p* = 0.0016, AUC = 0.59). In a multivariate logistic regression model, discharge from IRF to home with independence was predicted by change in 6MWT (*p* = 0.0007) but not hGS (*p* = 0.8075). Six‐month survival post‐rehabilitation was predicted by change in 6MWT (*p* = 0.0345) but not hGS (*p* = 0.9025) in a multivariate Cox proportional hazards model.

**Conclusions:**

Multiple analytical approaches to our data set demonstrate that changes in 6MWT are better associated with cachexia‐related outcomes and should be included in future cachexia studies.

## 1 Introduction

Cancer cachexia is a devastating muscle wasting disease that affects up to 80% of advanced cancer patients [1]. It is marked by significant weight loss and eventually progressive functional decline, which impairs quality of life and limits response to cancer therapy. Recent estimates suggest that 50%–90% of patients with cachexia will develop major disability [2]. Many attempts have been made to address cancer cachexia pharmacologically [3] and while there has been success in weight loss attenuation, no FDA‐approved treatment exists for cancer cachexia due to the inability of these drugs to improve the characteristic physical function decline in clinical trials. In previous trials, ‘physical function’ has been typically estimated by hand grip strength (hGS) [4], but recent reviews from Caiero et al. [5] and Ramage et al. [6] suggest hGS may not be an optimal measure of physical function, perhaps due to lack of sensitivity, statistical power or physiological relevance.

While hGS has been consistently shown to predict mortality in cancer [7], it has not been shown to be predictive or representative of the wide spectrum of functional capacity throughout the disease course. In fact, there is no single performance measure that comprehensively estimates total functional capacity. Multiple methods exist to determine functional independence, which is a measure of a patient's general mobility and ability to complete activities of daily living (ADLs), directly correlating with quality of life. Attempts to include more direct outcome measures of functional independence have included the use of alternative physical performances, such as the six‐minute walk test (6MWT), but there is not yet a consensus for what performance measures should be used as proxies for estimating cachexia‐related functional capacity [8, 9, 10]. In the interim, hGS continues to be used as a primary outcome in new studies of the cancer cachexia population [11].

Often referred to as the ‘functional vital sign’, gait speed results from the interplay of various body structures that necessitate the integration of postural control, lower body strength, cardiopulmonary endurance, proprioception, and vision functions [12]. Gait speed decline is consistently identified as a risk factor for disability, cognitive decline, admission to care facilities, falls, and mortality in frail and elderly individuals without cancer [13]. Moreover, gait speed is shown to be a relative measure of a patient's physical function and has potential to characterize function in cancer cachexia as it provides a continuous evaluation of walking pattern and behaviour [14]. The 6MWT is a validated measure of gait speed and endurance and has predictive value for survival in cancer populations [15, 16]. Given these findings, we compared the ability of 6MWT and hGS as proxy measures for physical function in cancer populations with cachexia.

Directly measuring functional independence is a resource‐intensive process. The inpatient rehabilitation setting offers a unique opportunity to investigate different outcome measures tested against gold standards for functional independence, as multiple functional measures are routinely collected by trained healthcare providers as part of the standard of care. Further, cachexia significantly impacts cancer patients receiving inpatient rehabilitation, and these patients have demonstrated significant functional improvements throughout the course of treatment [17]. Therefore, we leveraged our inpatient rehabilitation facility (IRF) population through retrospective cohort analysis to directly compare 6MWT and hGS to the functional and clinical outcomes of (1) functional independence, (2) discharge destination and (3) 6‐month survival. While recent reviews [10] suggest hGS and 6MWT are the most used physical function endpoints in cancer cachexia studies, to our knowledge, this is the first study to directly compare these two measures in their ability to approximate physical function. Our study demonstrates 6MWT is a substantially better predictor of multiple outcome measures than hGS in the cancer cachexia population. We suggest that gait‐based measures be implemented as primary outcome measures in cancer cachexia clinical trials intended to improve physical function.

## 2 Methods

### 2.1 Study Design

This human subjects study was approved by our Institutional Review Board of Northwestern University (STU0021683) on 19 January 2022 and received exempt status from consent procedures due to retrospective design.

**FIGURE 1:**
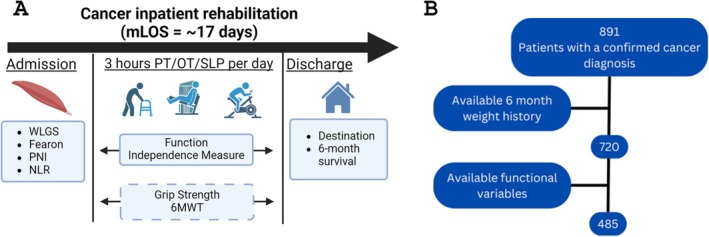
Inpatient rehabilitation course and study design. (A) Study design. Patients admitted to inpatient rehabilitation were retrospectively assessed for cachexia markers at the time of admission including Fearon et al. criteria, Weight Loss Grading Scale (WLGS), Prognostic Nutritional Index (PNI) and neutrophil‐to‐lymphocyte ratio (NLR). During the course of inpatient rehabilitation, patients received at least 3 h/day of therapy services between physical, occupational and speech language therapy. Measures of functional independence as well as measures of cognitive function (FIM) were collected at time of admission and discharge and were correlated with hand grip strength (hGS) and six‐minute walk test (6MWT) ambulation distance. Discharge outcomes were collected in the 6 months following discharge from the IRF, including discharge destination and survival outcomes. (B) CONSORT diagram depicting the exclusion process. Functional variables required for inclusion were admission and discharge 6MWT and functional independence measures.

This retrospective cohort study, depicted in Figure 1A, analysed cancer patient admissions to a free‐standing IRF from March 2017 to January 2022. Inclusion criteria were > 18 years old, prior oncologic care at an affiliated cancer centre and primary rehabilitation impairment related to cancer or its treatment. Patients lacking recorded weights within 6 months prior to IRF admission were excluded. Patients without available admission and discharge values for at least one measure of physical function were excluded.

Eight hundred ninety‐one encounters matched initial inclusion criteria. One hundred seventy‐one encounters were excluded because of a lack of available weight history. Two hundred thirty‐five encounters were excluded because of incomplete functional data, yielding a total of 485 encounters (Figure 1B). Of these 235 excluded encounters, 27 did not have a discharge Total Motor Score due to unplanned discharge to acute care, and 208 did not have available 6MWT data. As required by the Centers for Medicare and Medicaid Services and documented in the health record, all patients received a minimum of 3 h each day of a combination of physical (PT), occupational (OT) and language pathology (SLP) therapies for 5 days/week.

### 2.2 Data Collection

Data were collected from the acute care and IRF electronic medical records (EMRs) through manual review as well as through an automated electronic data extraction using the hospital system's proprietary Electronic Data Warehouse (EDW). Information detailing each subject's cancer diagnosis and treatment history, acute care and IRF courses, and demographic data were included. Body mass index (BMI) information (height, weight) was manually extracted from an affiliated cancer centre for the 6 months prior to IRF admission. Mortality data were collected from the acute care and IRF EMRs for up to 6 months after IRF discharge. Functional Independence Measure (FIM) scores or Section GG functional scores at admission and discharge were collected via the EDW and manual chart review.

The Charlson Comorbidity Index (CCI) considers 19 categories of medical conditions to calculate an overall score associated with mortality risk [18]. Subsequent publications provided scoring algorithms based on *International Classification of Diseases, Tenth Revision, Clinical Modification (ICD‐10‐CM)* codes in the United States [19]. Electronic and manual extraction of ICD‐10‐CM codes and comorbidity data were used to calculate each patient's total CCI score.

### 2.3 Cachexia Cohort Selection

Cachexia was defined primarily based on the Fearon et al. [20] diagnostic criteria for cancer cachexia: > 5% weight loss in 6 months or > 2% weight loss in 6 months with a BMI < 20 kg/m^2^. Unless otherwise specified, cachexia populations for analysis are defined based on this criterion. The Weight Loss Grading Scale (WLGS) serves as a secondary cachexia marker. Additional indicators of cachexia burden were the Prognostic Nutritional Index (PNI) and the neutrophil‐to‐lymphocyte ratio (NLR). The WLGS is an ordinal scale from zero to four that is derived from quintiles of BMI and percent weight loss over 6 months. Each increase in WLGS score corresponds to multiple squares on the BMI‐weight loss grid that are in a negative correlation to overall survival, as described in Martin et al. [21]. PNI (albumin level [g/L] + 5 × total lymphocyte count in 10^9^/μL) has been shown in several studies of cancer patients to be linked to malnutrition, cachexia and negative clinical outcomes [22]. The threshold for PNI was set to ≤ 40 or severe malnutrition [23]. NLR (neutrophil count per microliter/lymphocyte count per microliter) is correlated with poor survival outcomes and nutritional risk [24]. This threshold was set to > 6, which Xu et al. [25] determined to indicate significant physiologic stress.

### 2.4 Functional Outcome Measures

Admission and discharge values were collected for either the FIM or Section GG evaluation scores over the course of inpatient rehabilitation. FIM is a validated measure of functional status and includes 13 assessments of physical function [26]. A clinician scores each patient on a scale from 0 to 7, with 7 indicating complete independence and 0 indicating the need for total assistance. Until late 2018, FIM was the standard measurement tool used in IRFs to estimate motor and cognitive functional independence. Section GG of the current IRF‐Patient Assessment Instrument (IRF‐PAI) replaced FIM scores beginning in 2019 and is the current standard for motor function assessment. Section GG is a validated [27] assessment of motor function, including 25 sections. A clinician scored each patient on a scale from 1 to 6, with 6 indicating total independence and 1 indicating total dependence.

Due to this shift in function scoring from the FIM scale to the Section GG scale, a system was implemented to crosswalk these scores to allow for comparison of all encounters using an approach similar to prior functional measure crosswalks [28]. Briefly, FIM scores of 0 or 7 were converted into 1 or 6, respectively (the highest and lowest scores of the Section GG scale) and the ratio of the sum of all scores measured to the number of items measured was deemed ‘Total Motor Score’. This score reflects functional changes in ADLs, mobility, transfers and walking. Importantly, Section GG does not include a cognitive function assessment and, therefore, FIM scores continue to be used for cognitive scoring, including assessment of comprehension, problem solving and memory. Pre‐ and post‐crosswalk scores showed high correlation and similar predictive capacity for discharge independence (Table S1).

Secondary outcome measures included independence at discharge and survivorship 6 months post‐discharge. Independence at discharge was defined as discharge without the need for further inpatient care and was based on discharge destination (including home discharge without any home‐based therapy services, homebound with home‐based therapies, return to acute care or admission to a skilled nursing facility/long term acute care facility). The 6MWT and hGS dynamometry (Jamar Hydraulic Hand Dynamometer Performance Health #081028935/Jamar Smart Hand Dynamometer, Performance Health #081669928) were collected on admission and discharge by certified physical and occupational therapists as part of standard of care in our translational rehabilitation hospital. In all cases, the admission and discharge measurements were made by the subject's primary physical or occupational therapist.

### 2.5 Statistical Analysis

Statistical analyses were completed in GraphPad Prism 10.1 and IBM SPSS Statistics 26. Significance level (α) was set to 0.05 for all analyses. Descriptive data were analysed with chi‐squared tests for categorical variables and one‐way analysis of variance (ANOVA) for continuous variables. Paired analysis of pre‐ and post‐rehabilitation values was completed for 6MWT, hGS, Total Motor and Total Cognition values. Initial univariate linear regression was utilized for correlation of 6MWT and hGS with physical function gain in categories assessed with FIM/Section GG as well as the Total Motor Score. Gain was defined as the change in score between admission and discharge. Area under the curve (AUC) analysis for receiver‐operating characteristic (ROC) curves was performed to assess the predictive capacity of 6MWT and hGS on physical function gain. Sensitivity analysis was performed using Little's MCAR test for missing variables. Predictive mean matching (PMM) was then used in the multiple imputation of missing data. Recent studies suggest that this approach may be superior to standard methods in longitudinal/repeated measures settings^S1^. To preserve the distribution of the observed changes in Total Motor Score over time, which remains underdefined in the literature given its relatively new use, PMM was employed.

Within the cachexia population, as defined by the Fearon et al. consensus criteria, a stepwise approach was taken to developing unbiased, adjusted multivariate linear regression models to understand the competing relationship between 6MWT and hGS change with functional and clinical outcomes. Variables included were based on significance in univariate analyses and historically established clinical relevance. Total Motor Score gain was the dependent variable, and age, sex, increased disease burden (Stage 3, 4 or recurrence), cancer type (breast, primary intracranial, hematologic, genitourinary, gynecologic and common cachexia cancers [GI/pancreas/HPB/colon/lung]), cancer treatment history (chemotherapy, cancer related surgery, stem cell therapy and hormone therapy), 6MWT gain, hGS gain, length of stay and Total Motor Score at admission (baseline) were used as covariates (goodness‐of‐fit *r* ^2^ = 0.41, Cohen's *f* ^2^ = 0.69). Univariate and multivariate (Cox–Snell's *r* ^2^ = 0.24, Cohen's *f* ^2^ = 0.31) logistic regression was used to analyse binary relationships between changes in 6MWT and hGS with home independence upon discharge. Univariate and multivariate (pseudo *r* ^2^ = 0.16, Cohen's *f* ^2^ = 0.20) Cox proportional hazards regression was used to analyse changes in 6MWT and hGS with survivorship outcomes within 6 months (180 days) of discharge. Figure values are reported as mean ± SEM unless otherwise noted. While this was designed as a retrospective convenience sample, using G*Power 3.1.9.7, we calculated that our sample size would exceed power of 0.99 for any univariate analyses of 6MWT/hGS and motor function; and our sample size of 307 subjects would exceed power of 0.95 for any multivariate analyses that include up to 17 variables, assuming *f* ^2^ > 0.15.

## 3 Results

### 3.1 Demographics

**FIGURE 2:**
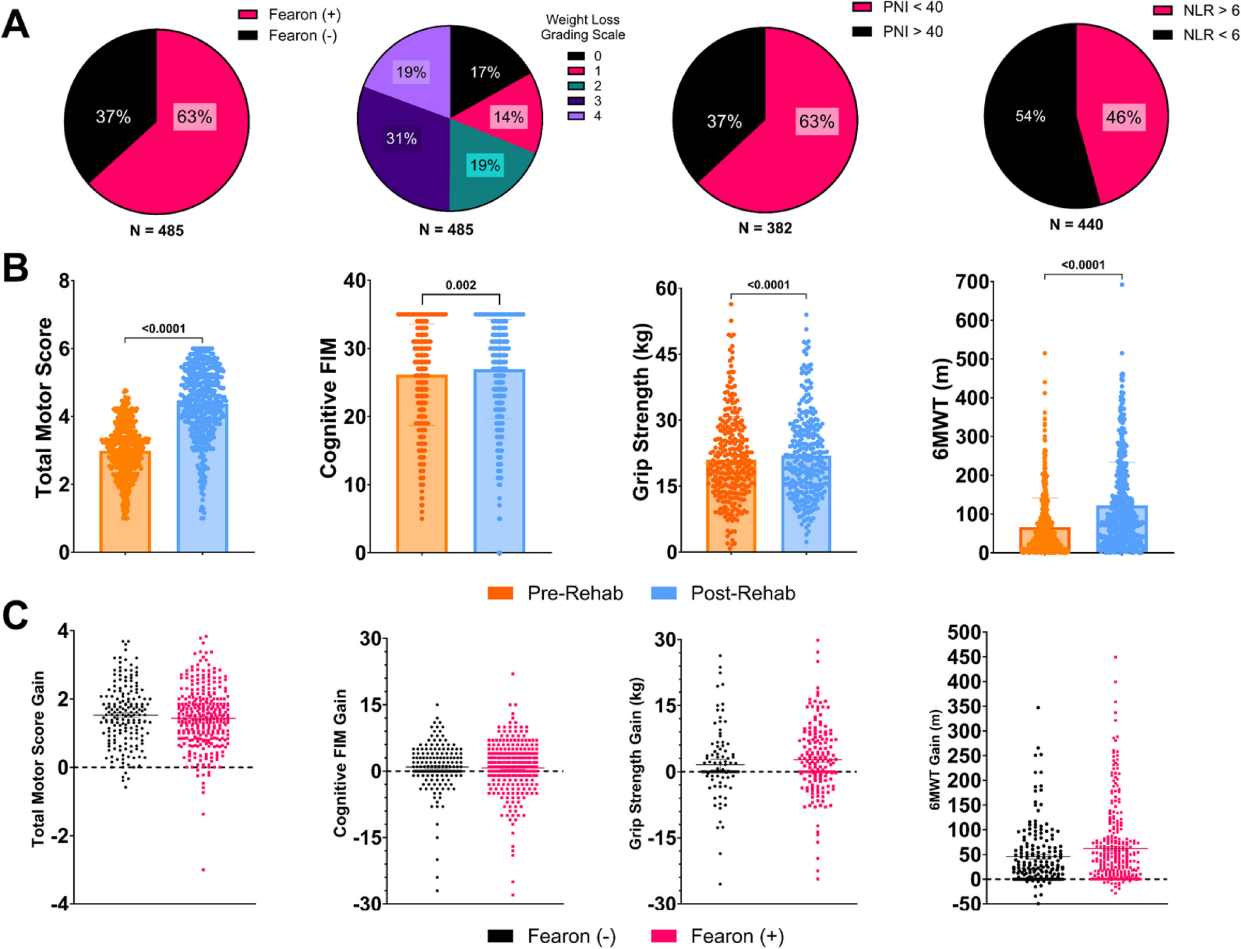
Muscle wasting distribution and change in functional measures during inpatient rehabilitation. (A) Distribution of cachexia markers, Fearon et al. criteria, WLGS or instances of cachexia burden (PNI, NLR) in cancer patients. *N* = number of patients with available data. (B) Change in outcome measures pre‐ and post‐ rehabilitation as seen in Total Motor Scores, cognitive FIM scores, hand grip strength (kg) and six‐minute walk test distance ambulated (m). Wilcoxon's test used to compare pre‐ and post‐repeated measures. (C) Gain in outcome measures as determined by the difference between pre‐ and post‐rehabilitation values separated by cachexia and no cachexia based on the Fearon et al. criteria. Mann–Whitney test used for comparison. *Note:* Panel CIII has four outliers and CIV has one outlier that is not included in the scale but was included in statistical analysis.

The prevalence of cachexia was 63% in this cohort of 485 patients admitted to our IRF for cancer‐related functional decline, similar to our prior report [29]. The mean length of stay was 17.88 ± 0.38 (SEM) days. We further classified this cachexia cohort with three more cachexia identifiers of interest (Figure 2A), WLGS, PNI and NLR. When classified by WLGS, 50% of patients had a high degree of cachexia (WLGS = 3 or 4, Table S2). Of the inpatients with available values, 63% met the serious malnutrition threshold for PNI and 46% met the NLR threshold for physiological stress. Notable demographic measures positively associated with cachexia were male sex (*p* = 0.005), hematologic cancer (*p* < 0.0001) and chemotherapy (*p* = 0.04) (Table 1). Measures negatively associated with cachexia were primary intracranial cancer (*p* = 0.014), prior cancer‐related surgery (*p* < 0.0001) and hormone therapy (*p* = 0.026). The combined group of lung and GI/pancreatic/hepatobiliary cancers trended towards a positive association with cachexia (*p* = 0.062) compared with the overall IRF cancer population. Age, IRF length of stay, increased disease burden and total CCI score had no significant association with cachexia incidence (all *p* > 0.20).

**TABLE 1:** Demographics of cancer patients receiving inpatient rehabilitation with hGS and 6MWT measured pre‐ and post‐rehabilitation.

Fearon criteria	(+)	(−)	Total	*p*‐value^a^
*n* (%)	307 (63%)	178 (37%)	485	
BMI^b^ [kg/m^2^]	25.52 ± 0.35	28.75 ± 0.47	26.70 ± 0.29	**< 0.0001**
6‐month % weight change^b^ [kg]	−11.92 ± 0.36	−0.13 ± 0.47	−7.59 ± 0.38	**< 0.0001**
Age^b^	62.59 ± 0.80	64.16 ± 1.04	63.16 ± 0.63	0.23
Gender (% male)	57.3%	43.8%	52.4%	**0.005**
Length of stay (days)^b^	17.89 ± 0.46	17.87 ± 0.66	17.88 ± 0.38	0.981
Cancer (*n*, %)				
Breast	16 (47%)	18 (53%)	34	0.063
Gastrointestinal	27 (73%)	10 (27%)	37	0.220
Genitourinary	15 (48%)	16 (52%)	31	0.085
Gynecologic	3 (43%)	4 (57%)	7	0.267
Head and neck	3 (50%)	3 (50%)	6	0.674
Hematologic	86 (78%)	24 (22%)	110	**< 0.0001**
Lung	34 (72%)	13 (28%	47	0.176
Musculoskeletal	8 (62%)	5 (38%)	13	1.00
Primary intracranial	99 (56%)	78 (44%)	177	**0.014**
Primary spine	5 (56%)	4 (44%)	9	0.731
Skin	12 (86%)	2 (14.3%)	14	0.095
Other	2 (66.7%)	1 (33.3%)	3	1.00
Multiple	3 (100%)	0 (0%)	3	0.302
Common cachexia cancers (GI/pancreas/HPB/colon/lung)^c^	61 (73%)	23 (27%)	84	**0.062**
Treatment history (*n*, %)				
Chemotherapy	205 (67%)	102 (33%)	307	**0.04**
Cancer related surgery	200 (57%)	149 (43%)	349	**< 0.0001**
Targeted therapy	95 (64%)	53 (36%)	148	0.838
Immunotherapy	41 (68%)	19 (32%)	60	0.475
Hormone therapy	12 (43%)	16 (57%)	28	**0.026**
Stem cell therapy	28 (72%)	11 (28%)	39	0.30
Radiation	156 (63%)	90 (37%)	246	1.00
Increased disease burden^d^	180 (65%)	98 (35%)	278	0.448
Total Charlson Comorbidity Index^b^	5.02 ± 0.17	4.93 ± 0.21	4.99 ± 0.13	0.749

*Note:* Items in bold indicate statistically significant results (*p* < 0.05).

^a^ Fisher's exact test (2‐sided), unless otherwise noted.

^b^ ANOVA comparison of means (demographic variable × cachexia status), reported as mean ± SEM.

^c^ Common cachexia cancers (gastrointestinal [GI], pancreas, hepatobiliary [HPB], colon, lung).

^d^ Increased disease burden indicates Stage 3/4 and/or the presence of metastasis.

### 3.2 Descriptive Analyses

Altogether, 485 patients had functional data available from admission and discharge. This cohort demonstrated significant improvement in Total Motor Score, cognitive FIM values, hGS and 6MWT measurements during inpatient rehabilitation (all *p* ≤ 0.002, Figure 2B). Importantly, a majority of both cachectic and non‐cachectic patients had positive gains in 6MWT, Total Motor Score and hGS (Figure 2C, Table S3). Among the subjects meeting Fearon et al. criteria, 307 had available 6MWT data and 196 had available hGS data.

### 3.3 Total Motor Score

We directly compared 6MWT and hGS as predictors of physical functional independence in cachectic patients, represented here as the ΔTotal Motor Score. Preliminary univariate linear analyses of Δ6MWT and ΔhGS against ΔTotal Motor Score in patients identified as cachectic using Fearon et al. criteria, WLGS, NLR or PNI revealed a correlation with Δ6MWT in all eight cachexia categories while ΔhGS only correlated with function in five (Table 2). Δ6MWT had significantly higher goodness‐of‐fit scores across all markers. A comparison of curves analysis between these Δ6MWT and ΔhGS univariate results also confirmed a significant difference in the slopes (*p* < 0.0001) across all cachexia markers.

**TABLE 2:** Univariate linear regression of Total Motor Score gain in cancer patients with muscle wasting as predicted by 6MWT vs. hGS gains.

	Slope	95% confidence interval (CI)	e^(Slope)^	e^(95% CI)^	*r* ^2^ value	*p*‐value^a^	Area under curve	ROC *p*‐value^b^
6MWT vs. Total Motor Score gain								
Fearon et al. criteria (+)	0.0043	0.0032–0.0055	1.004	1.003–1.006	0.148	**< 0.0001**	0.7742	**< 0.0001**
WLGS = 0	0.0050	0.0020–0.0079	1.005	1.002–1.008	0.125	**0.0011**	0.8415	**< 0.0001**
WLGS = 1	0.0050	0.0042–0.0108	1.005	1.004–1.011	0.236	**< 0.0001**	0.6373	**0.0054**
WLGS = 2	0.0032	0.0011–0.0053	1.003	1.001–1.005	0.090	**0.0037**	0.7559	**< 0.0001**
WLGS = 3	0.0040	0.0025–0.0055	1.004	1.002–1.006	0.157	**< 0.0001**	0.7841	**< 0.0001**
WLGS = 4	0.0056	0.0028–0.0083	1.006	1.003–1.008	0.148	**0.0001**	0.7812	**< 0.0001**
PNI < 40	0.0056	0.0040–0.0071	1.006	1.004–1.007	0.113	**< 0.0001**	0.7438	**< 0.0001**
NLR > 6	0.0053	0.0035–0.0071	1.005	1.004–1.007	0.147	**< 0.0001**	0.7617	**< 0.0001**
hGS vs. Total Motor Score gain								
Fearon et al. criteria (+)	0.0620	0.0255–0.0985	1.064	1.026–1.104	0.055	**0.001**	0.592	**0.0016**
WLGS = 0	0.1022	0.0308–0.1736	1.108	1.031–1.190	0.190	**0.0063**	0.5492	0.4608
WLGS = 1	0.0212	−0.0259 to 0.0682	1.021	0.974–1.071	0.022	0.3686	0.7219	**0.0007**
WLGS = 2	0.0232	−0.0527 to 0.0990	1.023	0.949–1.104	0.006	0.5432	0.7769	**< 0.0001**
WLGS = 3	0.0398	−0.0022 to 0.0819	1.041	0.998–1.085	0.038	0.0628	0.5156	0.7141
WLGS = 4	0.1499	0.0514–0.2484	1.162	1.053–1.282	0.136	**0.0035**	0.5853	0.104
PNI < 40	0.0937	0.0497–0.1378	1.098	1.051–1.148	0.102	**< 0.0001**	0.6161	**0.0004**
NLR > 6	0.0490	0.0134–0.0847	1.050	1.013–1.088	0.056	**0.0074**	0.5973	**0.0076**

*Note:* Items in bold indicate statistically significant results (*p* < 0.05).

^a^ Univariate linear regression.

^b^ Receiver‐operating characteristic (ROC) curve.

In our primary analysis of Fearon criteria positive cachectic subjects (*n* = 307), we employed a multivariate model to adjust for demographic data, cancer type, relevant cancer treatment history, comorbidities and baseline motor function (Table 3). With Δ6MWT and ΔhGS as additional independent variables, our multivariate model (goodness‐of‐fit *r* ^2^ = 0.41, Cohen's *f* ^2^ = 0.69) demonstrated gain in 6MWT distance ambulated (*p* < 0.0001), baseline Total Motor Score (*p* = 0.0016), sex (*p* = 0.0089), length of stay (*p* = 0.0016) and specific cancer types (primary intracranial [*p* = 0.01], hematologic [*p* = 0.0312]) were independently associated with Total Motor Score gain, while hGS gain did not have a significant influence on Total Motor Score gain (*p* = 0.084). Duplicate multivariate analysis with the removal of primary intracranial cancer as a potential driver of this effect showed no change in these results (data not shown).

**TABLE 3:** Multivariate linear analysis of functional independence scores in cancer patients with Fearon et al. criteria as predicted by 6MWT, hGS and selected clinically relevant variables.

Total Motor Score gain	Estimate	95% confidence interval (CI)	e^(Estimate)^	e^(95% CI)^	*p*‐value^a^
6MWT	0.004892	0.003399–0.00638	1.005	1.0034–1.0064	**< 0.0001**
hGS	0.02815	−0.003822 to 0.06013	1.029	0.9962–1.0620	0.084
Age	0.002948	−0.006599 to 0.01250	1.003	0.9934–1.0126	0.543
Sex	−0.3363	−0.5873 to −0.08525	0.714	0.5558–0.9183	**0.0089**
Breast	−0.03818	−0.2725 to 0.1962	0.963	0.7615–1.2168	0.7482
Primary intracranial	−0.8251	−1.450 to −0.1998	0.438	0.2346–0.8189	**0.01**
Hematologic	−0.4843	−0.9244 to −0.04427	0.616	0.3968–0.9567	**0.0312**
Genitourinary	0.0473	−0.4210 to 0.5156	1.048	0.6564–1.6746	0.8422
Common cachexia cancers (GI/pancreas/HPB/colon/lung)^b^	−0.2277	−0.7829 to 0.3275	0.796	0.4571–1.3875	0.4193
Chemotherapy	−0.2563	−0.6599 to 0.1473	0.774	0.5169–1.1587	0.2118
Cancer related surgery	−0.07831	−0.3288 to 0.1721	0.925	0.7198–1.1878	0.538
Stem cell therapy	0.08725	−0.2592 to 0.4337	1.091	0.7717–1.5430	0.6198
Hormone therapy	0.281	−0.1652 to 0.7272	1.324	0.8477–2.0693	0.2155
Increased disease burden^c^	−0.03818	−0.2725 to 0.1962	0.963	0.7615–1.2168	0.7482
Total CCI	−0.006715	−0.04938 to 0.03595	0.993	0.9518–1.0366	0.7565
Admit Total Motor Score	−0.2108	−0.3869 to −0.03480	0.810	0.6792–0.9658	**0.0016**
Length of stay (days)	0.02611	0.01007–0.04216	1.026	1.0101–1.0431	**0.0016**

*Note:* Items in bold indicate statistically significant results (*p* < 0.05).

Abbreviation: CCI = Charlson Comorbidity Index.

^a^ Multivariate linear regression, goodness‐of‐fit *r* ^2^ = 0.41, Cohen's *f* ^2^ = 0.69.

^b^ Common cachexia cancers (gastrointestinal [GI], pancreas, hepatobiliary [HPB], colon, lung).

^c^ Increased disease burden indicates Stage 3/4 and/or the presence of metastasis.

**FIGURE 3:**
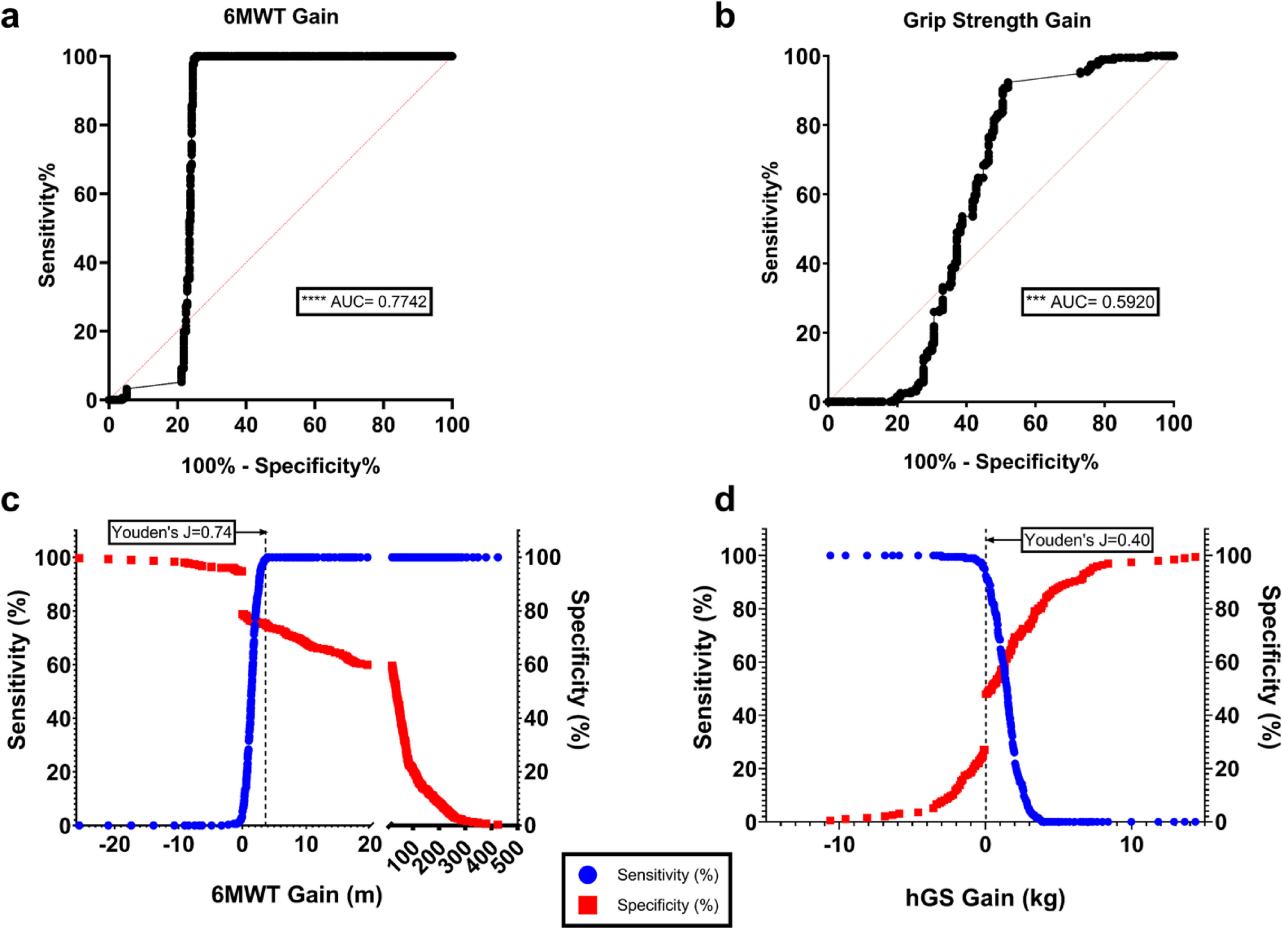
Receiver operating characteristic curves of 6MWT and hGS vs. Total Motor Scores in patients with cachexia. (A) Receiver‐operating characteristic (ROC) curve of patients with cachexia consensus criteria as determined by 6MWT gain vs. Total Motor Score gain. (B) ROC curve of patients with cachexia consensus criteria as determined by hGS gain vs. Total Motor Score gain. ROC curve evaluation reports significance if AUC > 0.5. (C) Representation of 6MWT gain and (D) hGS gain vs. sensitivity and specificity analysis with dashed line representing the threshold corresponding with the peak Youden's J for each functional measure. * = *p* < 0.05, *** = *p* < 0.01, **** = *p* < 0.0001.

Secondarily, we generated ROC curves (Figure 3A–B) for Δ6MWT × Total Motor Score and ΔhGS × Total Motor Score, which showed an AUC of 0.7742 (*p* < 0.0001) for Δ6MWT compared with only 0.592 (*p* = 0.0016) for ΔhGS, indicating a stronger positive and negative predictive capacity of the 6MWT compared with ΔhGS (Table 2). Δ6MWT above 4 m achieved 100% sensitivity, while ΔhGS reached 100% sensitivity in cases of decline (negative gain), suggesting complementary detection capability (Figure 3C,D). Further, threshold analysis showed that a Δ6MWT of 3.5–4.0 m (Youden's J = 0.74) had strong clinical utility for detecting Total Motor Score improvement, whereas ΔhGS thresholds of 0.07–0.2 kg were associated with a lower Youden's J of only 0.40, indicating weaker overall predictive ability (Figure S1).

To investigate the nature of the incomplete patient encounters, we conducted a missing value analysis of patients with muscle wasting and Total Motor Score gain values (*n* = 450). A total of 31.8% of these patients were missing Δ6MWT scores (*p* < 0.0001), and 56.4% were missing ΔhGS scores (*p* < 0.0001, Table S4). Based on the proportion of missing values, multiple imputations were generated using PMM (Table S5). The results of univariate linear regression were compared between each imputed dataset and our original analysis, which showed similar results for Δ6MWT (pooled *p* < 0.001, *r* ^2^ = 0.176, AUC = 0.767) and ΔhGS (pooled *p* < 0.001, *r* ^2^ = 0.061, AUC = 0.556), confirming that data missingness did not impact these correlative observations.

When separated by sex, Δ6MWT was stronger than ΔhGS for predicting ΔTotal Motor Score in both men (6MWT: AUC = 0.742, *p* < 0.0001; hGS: AUC = 0.59, *p* = 0.021) and women (6MWT: AUC = 0.818, *p* < 0.0001; hGS: AUC = 0.599, *p* = 0.024, Table S6). Importantly, linear regression of Δ6MWT and ΔhGS with Δcognitive FIM scores confirmed no significant correlation across all four cachexia identifiers, indicating cognitive function was not a major confounder in these observations (Table S7).

### 3.4 Discharge Independence

To further assess the link between these performance metrics and physical function, we assessed discharge outcomes as predicted by Δ6MWT and ΔhGS in cachectic patients. Level of care at discharge (i.e., requiring 24‐h supervision at a care facility or remaining homebound with home health services) is a major indicator of quality of life [30]. Adjusting for demographic data, cancer type, relevant cancer treatment history, comorbidities, length of stay and baseline motor function, multivariate logistic regression (Cox–Snell's *r* ^2^ = 0.24, Cohen's *f* ^2^ = 0.31) revealed a significant association between discharge independence and baseline Total Motor Score at admission (*p* < 0.0001), length of stay (*p* = 0.024) and gain in 6MWT distance ambulated (*p* = 0.0007), but not hGS gain (*p* = 0.8075) (Table 4).

**TABLE 4:** Logistic regression analysis of discharge independence of cancer patients with Fearon et al. cachexia consensus criteria as predicted by 6MWT, hGS and clinically relevant variables.

Discharge independence	Odds ratio estimate	95% CI	*p*‐value^a^
Univariate model			
6MWT	1.008	1.004–1.011	**< 0.0001**
hGS	1.041	0.9626–1.128	0.3156
Multivariate model			
6MWT	1.008	1.004–1.014	**0.0007**
hGS	1.012	0.9207–1.111	0.8075
Age	0.9949	0.9668–1.024	0.7251
Sex	1.241	0.5894–2.659	0.5732
Breast	0.5114	0.06602–3.285	0.4928
Primary intracranial	2.051	0.5741–7.575	0.2723
Hematologic	0.9963	0.2488–4.044	0.9958
Genitourinary	1.831	0.3163–9.652	0.4802
Common cachexia cancers (GI/pancreas/HPB/colon/lung)^b^	0.809	0.2437–2.687	0.7272
Chemotherapy	1.351	0.6339–2.924	0.4388
Cancer related surgery	1.401	0.4936–4.115	0.5297
Stem cell therapy	1.228	0.3143–4.693	0.7638
Hormone	0.5133	0.06053–3.999	0.5233
Increased disease burden^c^	1.174	0.5811–2.396	0.6551
Total CCI	0.905	0.7878–1.032	0.1454
Admit Total Motor Score	3.522	1.990–6.598	**< 0.0001**
Length of stay (days)	1.061	1.009–1.119	**0.024**

*Note:* Items in bold indicate statistically significant results (*p* < 0.05).

Abbreviation: CCI = Charlson Comorbidity Index.

^a^ Univariate and multivariate logistic regression, home discharge with independence includes discharge with orders for physician follow up, outpatient rehabilitation or complete independence, Cox–Snell's *r* ^2^ = 0.24, Cohen's *f* ^2^ = 0.31.

^b^ Common cachexia cancers (gastrointestinal [GI], pancreas, hepatobiliary [HPB], colon, lung).

^c^ Increased disease burden indicates Stage 3/4 and/or the presence of metastasis.

### 3.5 6‐Month Survival

Given that survival in the short‐term is a major factor in the consideration of appropriateness for participating in rehabilitation [31], data were collected from patients for up to 180 days (6 months) after discharge from rehabilitation. A multivariate Cox proportional hazards regression was performed to determine associations with survival within 180 days (6 months) of discharge, adjusting for the same variables as prior multivariate models (pseudo *r* ^2^ = 0.16, Cohen's *f* ^2^ = 0.20), Table 5). Age (*p* = 0.0072), total CCI score (*p* = 0.0005) were associated with negative 6‐month survival outcomes, while patients with a gain in 6MWT had significantly decreased odds of death (*p* = 0.0345). There was no significant correlation between hGS and survival outcome (*p* = 0.9025).

**TABLE 5:** Cox regression of 6‐month survival outcomes in cancer patients with Fearon et al. cachexia consensus criteria as predicted by 6MWT, hGS and selected clinically relevant variables.

Death within 180 days of discharge	Hazard ratio estimate	95% CI	*p*‐value^a^
Univariate model			
6MWT	0.996	0.9926–0.9989	**0.0126**
hGS	0.9734	0.9099–1.039	0.4282
Multivariate model			
6MWT	0.9956	0.9912–0.9995	**0.0345**
hGS	1.005	0.9313–1.081	0.9025
Age	1.033	1.010–1.059	**0.0072**
Sex	1.327	0.7421–2.442	0.3496
Breast	0.7019	0.1789–2.476	0.5926
Primary intracranial	1.937	0.7292–5.322	0.1905
Hematologic	0.8863	0.3621–2.206	0.792
Genitourinary	0.5149	0.1283–1.619	0.2963
Common cachexia cancers (GI/pancreas/HPB/colon/lung)^b^	0.5033	0.1961–1.238	0.1396
Chemotherapy	0.9777	0.5479–1.796	0.9404
Cancer related surgery	1.298	0.6110–2.779	0.4978
Stem cell therapy	1.53	0.5141–4.084	0.4133
Hormone therapy	2.752	0.6697–9.446	0.1263
Increased disease burden^c^	0.8895	0.5257–1.513	0.6626
Total CCI	1.187	1.078–1.309	**0.0005**
Admit Total Motor Score	1.463	0.9743–2.239	0.0729
Length of stay (days)	0.9775	0.9407–1.015	0.2387

*Note:* Items in bold indicate statistically significant results (*p* < 0.05).

Abbreviation: CCI = Charlson Comorbidity Index.

^a^ Univariate and multivariate Cox hazard's ratio analysis, pseudo *r* ^2^ = 0.16, Cohen's *f* ^2^ = 0.20.

^b^ Common cachexia cancers (gastrointestinal [GI], pancreas, hepatobiliary [HPB], colon, lung).

^c^ Increased disease burden indicates Stage 3/4 and/or the presence of metastasis.

## 4 Discussion

In this study of cancer cachexia patients undergoing rehabilitation, we examined the relationship between multiple physical performance measures and key functional and clinical outcomes using multivariate analysis. Our inpatient rehabilitation cancer population was prominently affected by cachexia measured by multiple markers, yet made significant gains in functional independence, cognitive function, hGS and 6MWT measurements. Change in 6MWT emerged as a more reliable predictor of physical function, independence upon discharge and survival within 6 months post‐discharge compared with changes in hGS. These findings held true across a variety of cachexia identifiers. The utility of 6MWT as an excellent functional outcome measure was established through multivariate analyses, accounting for variables such as cancer stage and type, comorbidities, baseline physical function, age, sex, length of stay and BMI.

Multiple attempts have been made to develop pharmacologic therapies that reverse the effects of cachexia, but none received FDA approval in the United States. For example, clinical trials of anamorelin showed improvement in lean body mass, but approval was not granted because of a lack of hGS improvement [4]. Recent studies suggest a more direct measurement of functional independence may be favourable for clinical trials with cachexia patients [9, 32]. A newly published Phase 2 trial of ponsegromab, a GDF‐15 inhibitor, implements this approach by showing improvement in digital measures of daily physical activity in cancer cachexia patients, as well as increases in weight gain, appetite and skeletal muscle mass [33]. This gain in gait‐based physical activity is promising and may translate to clinically important functional outcomes such as ADLs. However, the search to find a physical performance metric that objectively determines a patient's functional ability and quality of life is necessary to determine the efficacy of future trials. To our knowledge, this is the first time that hGS and 6MWT have been tested and compared as indicators of functional independence in cancer populations with and without cachexia.

Our study demonstrates that hGS alone does not correlate strongly with functional independence, similar to the findings of Anderson et al. who showed hGS lacks broad sensitivity as a functional outcome in cachectic populations [34]. Because of this, alternative metrics have been proposed in recent reviews as a proxy for physical function, including the 6MWT, timed sit‐to‐stand, stair climbing power and various patient‐reported outcomes [5]. Interestingly, prior studies of age‐related sarcopenia suggest that gait speed is an early indicator of clinical decline [35, 36]. Given our findings, we propose that because gait reflects the synchronized function of several muscle groups and body systems, it may be a more sensitive and specific functional measure compared with hGS, which only involves distal limb muscles affected relatively late in disease progression. Walking biomechanically requires high energetic demand from the lower extremity proximal muscle groups, including both gluteal and quadriceps muscles, which are the largest muscles in nearly all mammals. Larger muscles are likely to be more affected by wasting mechanisms before distal, smaller muscles because they serve as a larger protein depot for the body. These data are also similar to our recent pre‐clinical work showing that: (1) gait speed changes are more specific to biological components of cachexia, while hGS is only linked to tumour‐related components; and (2) gait changes precede grip strength changes in the natural history of the development of cachexia [37].

A major goal of cancer rehabilitation is to improve quality of life by maximizing physical functional independence after the debilitating effects of the disease and its treatment. Home discharge with independence is an important factor when considering increased quality of life. IRF cancer patients with higher levels of functional independence at discharge have a greater likelihood of avoiding rehospitalization or institutionalization [31]. We expand upon this idea by comparing the predictive capacity of 6MWT and hGS gains in discharge outcomes, providing evidence that 6MWT gain better estimates improvements in physical function and discharge independence in both cachectic and non‐cachectic populations. Thus, we propose 6MWT has more utility as an all‐purpose physical performance measure for predicting functional independence in the real world setting after undergoing inpatient rehabilitation.

Cachexia is a serious condition that causes more than 30% of cancer‐related deaths [38], making efforts to identify effective predictors of quality of life and adverse outcomes of paramount importance. Here, we showed gains in 6MWT were associated with decreased odds of death within 6 months post‐discharge, highlighting its potential use in prognostic assessments. Historically, hGS has been tied to mortality in a wide variety of populations, such as aging [39], stroke recovery [40] and cancer [5, 7, 11]. However, our data did not demonstrate such an association 6 months post‐rehabilitation when included in a comparative multivariate model.

The inverse sensitivity relationship between 6MWT and hGS indicates that these two performance metrics have distinct value in monitoring recovery or decline over time. As we previously reported [32], combinatorial approaches will be necessary for future cachexia clinical trials. hGS change was more sensitive in the negative range compared with the positive range, suggesting it is a strong predictor of decline but not as strong at predicting recovery. This notion offers a potential explanation for why prior trials were unable to show recovery using this metric alone [3, 4]. In contrast, 6MWT showed a broader range for sensitivity that was only in the positive range of change, indicating that it is better suited to detecting recovery rather than decline. Thus, in future studies, we suggest that hGS should be used for screening and monitoring the likelihood of decline, as it is in sarcopenia criteria [5, 7], but 6MWT (or similar gait measures) should be used to estimate functional recovery.

Limitations include the single‐site nature of the study, conducted at an oncology centre and affiliated rehabilitation facility between 2017 and 2022. Selective admission to our IRF—based on functional decline during an acute hospital stay and nonclinical factors like geography, socioeconomic status and insurance—limits generalizability. The cohort's diversity in cancer type makes it difficult to account for variations in stage, treatment and response, though we categorized these factors and adjusted for them in multivariate models. Models with and without CNS tumours showed no significant difference, but ultimately the diverse and retrospective nature of the cohort limits our ability to account for all covariates. Other medical sources of impairment, such as neuropathy, may affect functional decline. The retrospective design limits our ability to correct for data missingness and selection bias, and also control covariates, and draw causal conclusions, though we attempted to mitigate this with a large cohort and continuity of care. Our approach using PMM to assess the impact of data missingness was dependent on having sufficient, well‐distributed donor values. While few studies have rigorously evaluated these methods due to limited theory beyond simulations^S1^, the low variability between imputations and the original dataset (Table S6) suggests PMM was a reasonable choice for handling missing data.

Further, this cohort may not represent less functionally impaired cachexia patients earlier in the disease spectrum, who may require different performance metrics. This underscores the need for a comparative study of multiple metrics in outpatient settings with lower severity cachexia patients to determine the best measure of post‐discharge functional capacity. Our study does not assess the interaction between functional change and muscle mass/body weight recovery due to the short IRF stay. Future prospective and longitudinal studies in cancer populations receiving outpatient rehabilitation are necessary to elucidate relationships between muscle mass and functional recovery. While this retrospective study demonstrates 6MWT to be a better proxy measure of physical function improvements compared with hGS, our relatively low *r* ^2^ values suggest much prospective work remains to determine the minimum combinations of tests needed to comprehensively predict functional capacity related to cachexia.

## 5 Conclusion

In summary, our multivariate analyses demonstrate that 6MWT is a strong proxy outcome for cachexia‐related functional outcomes. Secondary analysis then shows that 6MWT is a sensitive marker of functional recovery during cachexia. Future prospective studies in the outpatient setting should explore the combinatorial role of 6MWT, hGS and other metrics in detecting cachexia‐related functional recovery and decline.

## Ethics Statement

The human subjects study was approved by our Institutional Review Board (STU00216813) on 19 January 2022 and received exempt status due to its retrospective design.

## Conflicts of Interest

The authors declare no conflicts of interest.

## Supporting information


**Table S1.** Validation of cross‐walking method to combine multiple functional assessments through logistic and linear regression.
**Table S2.** Demographics of cancer patients receiving inpatient rehabilitation with grip strength and 6MWT measured pre and post rehabilitation, separated by WLGS.
**Table S3.** Outcomes of cancer patients with and without muscle wasting receiving inpatient rehabilitation measured pre‐ and post‐rehabilitation.
**Table S4.** Missing variable analysis of 6MWT and hGS in cancer patients with Fearon et al. criteria and Total Motor Score gain values.
**Table S5.** Univariate linear regression of Total Motor Score gain in cancer patients Fearon et al. criteria as predicted by 6MWT vs. hGS gains with multiple imputations of missing functional outcomes.
**Table S6.** Univariate linear regression of Total Motor Score gain in cancer patients with Fearon et al. criteria as predicted by 6MWT vs. hGS gains, separated by sex.
**Table S7.** Univariate linear regression of cognitive FIM score gain in cancer patients with muscle wasting as predicted by 6MWT vs. hGS gains.
**Figure S1.** Youden’s J calculated from ROC‐derived sensitivity and specificity values for (a) 6MWT gain and (b) hGS gain in their ability to predict Total Motor Score gain.
