# Supplementary material for: Six‐Minute Walk Test Is Superior to Grip Strength as a Marker of Functional Recovery During Cancer Cachexia Rehabilitation
Source: J Cachexia Sarcopenia Muscle. 2025 Jul 29;16(4):e70024. doi: 10.1002/jcsm.70024 (PMC12304731; doi:10.1002/jcsm.70024)
Supplement: Supplementary file 1 — Table S1. Validation of cross‐walking method to combine multiple functional assessments through logistic and linear regression. Table S2. Demographics of cancer patients receiving inpatient rehabilitation with grip strength and 6MWT measured pre and post rehabilitation, separated by WLGS. Table S3. Outcomes of cancer patients with and without muscle wasting receiving inpatient rehabilitation measured pre‐ and post‐rehabilitation. Table S4. Missing variable analysis of 6MWT and hGS in cancer patients with Fearon et al. criteria and Total Motor Score gain values. Table S5. Univariate linear regression of Total Motor Score gain in cancer patients Fearon et al. criteria as predicted by 6MWT vs. hGS gains with multiple imputations of missing functional outcomes. Table S6. Univariate linear regression of Total Motor Score gain in cancer patients with Fearon et al. criteria as predicted by 6MWT vs. hGS gains, separated by sex. Table S7. Univariate linear regression of cognitive FIM score gain in cancer patients with muscle wasting as predicted by 6MWT vs. hGS gains. Figure S1. Youden’s J calculated from ROC‐derived sensitivity and specificity values for (a) 6MWT gain and (b) hGS gain in their ability to predict Total Motor Score gain. [file JCSM-16-e70024-s001.docx]

**Supplemental Tables**

**Table S1: Validation of cross-walking method to combine multiple functional assessments through logistic and linear regression.**

|  | Functional Assessment Motor Score Gain vs Cross-walked Total Motor Score gain | | Cross-walked Total Motor Score Gain vs Functional Assessment Total Motor Score gain | | Functional Assessment Motor Score Gain vs Discharge Destination | | | Cross-walked Total Motor Score Gain vs Discharge Destination | | |
| --- | --- | --- | --- | --- | --- | --- | --- | --- | --- | --- |
| Standard Physical Function Assessment (Years implemented) | **R^2^** | **p-value^a^** | **R^2^** | **p-value^a^** | **R^2^** | **p-value^b^** | **Area under ROC curve** | **R^2^** | **p-value^b^** | **Area under ROC curve** |
| FIM (<2019) | 1.00 | **<0.0001** | 1.00 | **<0.0001** | 0.314 | **<0.0001** | 0.8623 | 0.314 | **<0.0001** | 0.7063 |
| Section GG (>2019) | 0.705 | **<0.0001** | 0.705 | **<0.0001** | 0.310 | **<0.0001** | 0.8661 | 0.246 | **<0.0001** | 0.8263 |

^a^Univariate linear regression

^b^Univariate logistic regression

ROC= Receiver Operating Characteristic, FIM= Functional Independence Measure, Section GG= of the current IRF-Patient Assessment Instrument (IRF-PAI)

Items in bold indicate statistically significant results (p <0.05)

**Table S2: Demographics of cancer patients receiving inpatient rehabilitation with grip strength and 6MWT measured pre and post rehabilitation, separated by WLGS.**

| **WLGS** | **0** | **1** | **2** | **3** | **4** | **Total** | **p-value^a^** |
| --- | --- | --- | --- | --- | --- | --- | --- |
| **n (%)** | 82 (17%) | 69 (14%) | 92 (19%) | 148 (31%) | 94 (19%) | 485 |  |
| **BMI^b^ [kg/m^2^]** | 2.33 ± 0.84 | -2.54 ± 0.53 | -5.91 ± 0.24 | -10.41 ± 0.43 | -17.17 ± 0.69 | -7.59 ± 0.38 | **<0.0001** |
| **6-Month % Weight Change^b^ [kg]** | 31.31 ± 0.54 | 30.97 ± 0.80 | 27.31 ± 0.59 | 25.38 ± 0.44 | 21.04 ± 0.31 | 26.70 ± 0.29 | **<0.0001** |
| **Age^b^** | 65.19 ± 1.54 | 60.09 ± 1.56 | 63.45 ± 1.47 | 60.62 ± 1.25 | 66.81 ± 1.16 | 63.16 ± 0.63 | **0.004** |
| **Gender (% male)** | 43.90% | 44.90% | 47.80% | 58.80% | 59.60% | 52.40% | **0.060** |
| **Length of stay (days)^b^** | 19.18 ± 1.05 | 18.22 ± 1.16 | 16.96 ± 0.72 | 17.80 ± 0.61 | 17.53 ± 0.89 | 17.88 ± 0.377 | 0.487 |
|  |  |  |  |  |  |  |  |
| **Cancer (n, %)** |  |  |  |  |  |  |  |
| **Breast** | 11 (32%) | 4 (12%) | 8 (23%) | 6 (18%) | 5 (15%) | 34 | **0.088** |
| **Gastrointestinal** | 3 (8%) | 5 (14%) | 7 (19%) | 13 (35%) | 9 (24%) | 37 | 0.621 |
| **Genitourinary** | 6 (19%) | 5 (16%) | 8 (26%) | 9 (29%) | 3 (10%) | 31 | 0.618 |
| **Gynecologic** | 1 (14%) | 0 (0%) | 2 (29%) | 3 (43%) | 1 (14%) | 7 | 0.766 |
| **Head & Neck** | 1 (17%) | 2 (17%) | 2 (33%) | 3 (33%) | 0 (0.0%) | 6 | 0.758 |
| **Hematologic** | 8 (7%) | 12 (11%) | 11 (10%) | 39 (36%) | 40 (36%) | 110 | **<0.0001** |
| **Lung** | 8 (17%) | 2 (4%) | 9 (19%) | 14 (30%) | 14 (30%) | 47 | 0.161 |
| **Musculoskeletal** | 2 (15%) | 3 (23%) | 4 (31%) | 3 (23%) | 1 (8%) | 13 | 0.572 |
| **Primary intracranial** | 40 (23%) | 33 (19%) | 38 (21%) | 50 (28%) | 16 (9%) | 177 | **<0.0001** |
| **Primary Spine** | 1 (11%) | 3 (33%) | 1 (11%) | 2 (22%) | 2 (22%) | 9 | 0.545 |
| **Skin** | 0 (0%) | 1 (7%) | 1 (7%) | 7 (50%) | 5 (37.5%) | 14 | 0.1 |
| **Other** | 1 (33%) | 0 (0%) | 1 (33%) | 1 (33%) | 0(0.0%) | 3 | 0.767 |
| **Multiple** | 0 (0%) | 0 (0%) | 0 (0%) | 1 (33%) | 2 (67%) | 3 | 0.287 |
| **Common cachexia cancers (GI/Pancreas/HPB/Colon/ Lung)^c^** | 11 (13%) | 7 (8%) | 16 (19%) | 27 (32%) | 23 (28%) | 84 | 0.147 |
|  |  |  |  |  |  |  |  |
| **Treatment History (n, %)** |  |  |  |  |  |  |  |
| **Chemotherapy** | 41 (13%) | 42 (14%) | 64 (21%) | 97 (32%) | 63 (20%) | 307 | 0.065 |
| **Cancer related surgery** | 74 (21%) | 55 (16%) | 72 (21%) | 103 (29%) | 45 (13%) | 349 | <0.0001 |
| **Targeted therapy** | 18 (12%) | 20 (14%) | 33 (22%) | 44 (30%) | 33 (22%) | 148 | 0.274 |
| **Immunotherapy** | 9 (15%) | 8 (13%) | 10 (17%) | 21 (35%) | 12 (20%) | 60 | 0.933 |
| **Hormone therapy** | 9 (32.1%) | 3 (10.7%) | 6 (21.4%) | 7 (25.0%) | 3 (10.7%) | 28 | 0.208 |
| **Stem cell therapy** | 1 (3%) | 7 (18%) | 5 (13%) | 13 (33%) | 13 (33%) | 39 | 0.029 |
| **Radiation** | 40 (16%) | 37 (15%) | 51 (21%) | 75 (31%) | 43 (17%) | 246 | 0.716 |
|  |  |  |  |  |  |  |  |
| **Increased disease burden^d^** | 41 (15%) | 41 (15%) | 59 (21%) | 86 (31%) | 51 (18%) | 278 | 0.398 |
| **Total Charleson Comorbidity Index^b^** | 4.51 ± 2.39 | 5.33 ± 3.25 | 4.71 ± 2.57 | 5.07 ± 3.02 | 5.30 ± 3.01 | 4.99 ±2.88 | 0.257 |

^a^Pearson’s chi-squared analysis, unless otherwise noted

^b^ANOVA comparison of means (demographic variable x cachexia status), reported as mean ± SEM

^c^Common cachexia cancers (Gastrointestinal (GI), Pancreas, Hepatobiliary (HPB), Colon, Lung)

^d^Increased disease burden indicates stage 3/4 and/or the presence of metastasis.

Items in bold indicate statistically significant results (p <0.05)

**Table S3: Outcomes of cancer patients with and without muscle wasting receiving inpatient rehabilitation measured pre- and post- rehabilitation.**

| Fearon Criteria | (+) | | | | (-) | | | | Total | | | |
| --- | --- | --- | --- | --- | --- | --- | --- | --- | --- | --- | --- | --- |
| Outcome (mean ± SEM) | **n** | **Pre-** | **Post-** | **p-value^a^** | **n** | **Pre-** | **Post-** | **p-value^a^** | **n** | **Pre-** | **Post-** | **p-value^a^** |
| Total Motor Score | 307 | 2.983 ± .045 | 4.33 ± .059 | **<0.0001** | 178 | 3.00 ± .059 | 4.44 ± .079 | **<0.0001** | 485 | 2.99 ± .036 | 4.37 ± .047 | **<0.0001** |
| Cognitive FIM | 298 | 26.16 ± .438 | 26.89 ± .420 | **0.028** | 172 | 26.10 ± .555 | 27.03 ± .563 | **0.023** | 470 | 26.14 ± .343 | 26.94 ± .336 | **0.002** |
| 6MWT (m) | 307 | 62.37 ± 3.90 | 124.37 ± 6.50 | **<0.0001** | 178 | 73.78 ± 6.34 | 119.13 ± 8.10 | **<0.0001** | 485 | 66.56 ± 3.40 | 122.42 ± 5.07 | **<0.0001** |
| hGS (kg) | 196 | 20.09 ± .658 | 21.31 ± .663 | **<0.0001** | 96 | 22.81 ± 1.01 | 23.53 ± 1.05 | 0.19 | 292 | 20.99 ± .575 | 22.04 ± .566 | **<0.0001** |

^a^Paired-samples t-test, two-tailed

Items in bold indicate statistically significant results (p <0.05)

**Table S4: Missing variable analysis of 6MWT and hGS in cancer patients with Fearon *et al.* criteria and Total Motor Score gain values**

| Functional Outcome | Total (n) | Patients with functional outcome value (n) | Patients without functional outcome value (n, %) | Little's MCAR p-value |
| --- | --- | --- | --- | --- |
| 6MWT gain | 450 | 307 | 143(31.8%) | **<0.0001** |
| hGS gain | 450 | 196 | 254(56.4%) | **<0.0001** |

Items in bold indicate statistically significant results (p <0.05)

**Table S5: Univariate linear regression of Total Motor Score gain in cancer patients Fearon *et al.* criteria as predicted by 6MWT vs hGS gains with multiple imputations of missing functional outcomes**

|  | |  | Slope | 95% Confidence Interval (CI) | e^(Slope)^ | e^(95% CI)^ | r^2^ value | p-value^a^ | Area Under Curve | ROC p-value^b^ |
| --- | --- | --- | --- | --- | --- | --- | --- | --- | --- | --- |
| 6MWT gain vs Total Motor Score gain | | | |  |  |  |  |  |  |  |
| Original | | | 0.0043 | 0.0032 to 0.0055 | 1.004 | 1.003 to 1.006 | 0.148 | **<0.0001** | 0.7742 | **<0.0001** |
| Imputation #1 | | | 0.0053 | 0.0043 to 0.0064 | 1.005 | 1.004 to 1.006 | 0.176 | **<0.001** | 0.7668 | **<0.0001** |
| Imputation #2 | | | 0.0053 | 0.0043 to 0.0064 | 1.005 | 1.004 to 1.006 | 0.176 | **<0.001** | 0.7668 | **<0.0001** |
| Imputation #3 | | | 0.0053 | 0.0043 to 0.0064 | 1.005 | 1.004 to 1.006 | 0.176 | **<0.001** | 0.7668 | **<0.0001** |
|  | **Pooled** | | 0.0053 | 0.0043 to 0.0064 | 1.005 | 1.004 to 1.006 | 0.176 |  | 0.7668 |  |
|  | | |  |  |  |  |  |  |  |  |
| hGS gain vs Total Motor Score gain | | | |  |  |  |  |  |  |  |
| Original | | | 0.062 | 0.0255 to 0.0985 | 1.064 | 1.0626 to 1.104 | 0.055 | **0.001** | 0.592 | **0.0016** |
| Imputation #1 | | | 0.074 | 0.0517 to 0.0956 | 1.077 | 1.053 to 1.100 | 0.089 | **<0.001** | 0.5777 | **<0.0001** |
| Imputation #2 | | | 0.056 | 0.0339 to 0.079 | 1.058 | 1.034 to 1.082 | 0.051 | **<0.001** | 0.5629 | **0.0011** |
| Imputation #3 | | | 0.058 | 0.0346 to 0.0804 | 1.060 | 1.035 to 1.084 | 0.052 | **<0.001** | 0.5325 | 0.0912 |
| Imputation #4 | | | 0.042 | 0.018 to 0.0657 | 1.043 | 1.018 to 1.068 | 0.026 | **<0.001** | 0.5262 | 0.1738 |
| Imputation #5 | | | 0.077 | 0.0543 to 0.1002 | 1.080 | 1.056 to 1.105 | 0.089 | **<0.001** | 0.5801 | **<0.0001** |
| Pooled | | | 0.061 | 0.0157 to 0.1052 | 1.063 | 1.016 to 1.111 | 0.061 |  | 0.5559 |  |

^a^Univariate linear regression

^b^Receiver operating characteristic (ROC) curve

Items in bold indicate statistically significant results (p <0.05)

**Table S6: Univariate linear regression of Total Motor Score gain in cancer patients with Fearon *et al.* criteria as predicted by 6MWT vs hGS gains, separated by sex.**

|  | **Slope** | **95% Confidence Interval (CI)** | **e^(Slope)^** | **e^(95% CI)^** | **r^2^ value** | **p-value^a^** | **Area Under Curve** | **ROC p-value^b^** |
| --- | --- | --- | --- | --- | --- | --- | --- | --- |
| **6MWT gain (n)** | |  |  |  |  |  |  |  |
| Male (176) | 0.004 | 0.0029 to 0.0058 | 1.004 | 1.003 to 1.006 | 0.1702 | **<0.0001** | 0.742 | **<0.0001** |
| Female (131) | 0.004 | 0.0020 to 0.0060 | 1.004 | 1.002 to 1.006 | 0.1124 | **<0.0001** | 0.818 | **<0.0001** |
| **hGS gain (n)** | | |  |  |  |  |  |  |
| Male (109) | 0.055 | 0.0111 to 0.0992 | 1.057 | 1.011 to 1.104 | 0.054 | **0.015** | 0.590 | **0.021** |
| Female (87) | 0.070 | 0.0056 to 0.1339 | 1.072 | 1.006 to 1.143 | 0.052 | **0.033** | 0.599 | **0.024** |

^a^Univariate linear regression

^b^Receiver operating characteristic (ROC) curve

Items in bold indicate statistically significant results (p <0.05)

**Table S7: Univariate linear regression of Cognitive FIM Score gain in cancer patients with muscle wasting as predicted by 6MWT vs hGS gains.**

|  | **Slope** | **95% Confidence Interval (CI)** | **e^(Slope)^** | **e^(95% CI)^** | **p-value^a^** |
| --- | --- | --- | --- | --- | --- |
| **6MWT vs FIM Cognitive Score** | |  |  |  |  |
| **Fearon *et al.* criteria (+)** | 0.0000 | -0.0082 to 0.0081 | 1.000 | 0.992 to 1.008 | 0.9871 |
| **WLGS=0** | 0.0035 | -0.00796 to 0.0306 | 1.004 | 0.992 to 1.031 | 0.2457 |
| **WLGS=1** | 0.0019 | -0.0113 to 0.0235 | 1.002 | 0.989 to 1.024 | 0.4884 |
| **WLGS=2** | 0.0023 | -0.0067 to 0.0219 | 1.002 | 0.993 to 1.022 | 0.2943 |
| **WLGS=3** | -0.0012 | -0.0159 to 0.0083 | 0.999 | 0.984 to 1.008 | 0.5329 |
| **WLGS=4** | 0.0017 | -0.01254 to 0.02342 | 1.002 | 0.988 to 1.024 | 0.5495 |
| **PNI < 40** | 0.0018 | -0.0033 to 0.0152 | 1.002 | 0.997 to 1.015 | 0.2094 |
| **NLR > 6** | 0.0014 | -0.0060 to 0.0149 | 1.001 | 0.994 to 1.015 | 0.4032 |
|  |  |  |  |  |  |
| **hGS vs FIM Cognitive Score** | |  |  |  |  |
| **Fearon *et al.* criteria (+)** | -0.0181 | -0.2692 to 0.1896 | 0.982 | 0.764 to 1.209 | 0.7325 |
| **WLGS=0** | 0.0038 | -0.3321 to 0.3488 | 1.004 | 0.717 to 1.417 | 0.9604 |
| **WLGS=1** | -0.0865 | -0.3677 to -0.0138 | 0.917 | 0.692 to 0.986 | 0.0354 |
| **WLGS=2** | -0.0294 | -0.5421 to 0.4125 | 0.971 | 0.582 to 1.511 | 0.7868 |
| **WLGS=3** | -0.0587 | -0.4147 to 0.1560 | 0.943 | 0.661 to 1.169 | 0.3700 |
| **WLGS=4** | 0.2142 | -0.1726 to 1.117 | 1.239 | 0.841 to 3.056 | 0.1480 |
| **PNI < 40** | -0.0206 | -0.2621 to 0.1713 | 0.980 | 0.769 to 1.187 | 0.6796 |
| **NLR > 6** | -0.0667 | -0.3345 to 0.0406 | 0.935 | 0.716 to 1.041 | 0.1234 |

^a^Univariate linear regression

Items in bold indicate statistically significant results (p <0.05)

**Supplemental Figures**

**
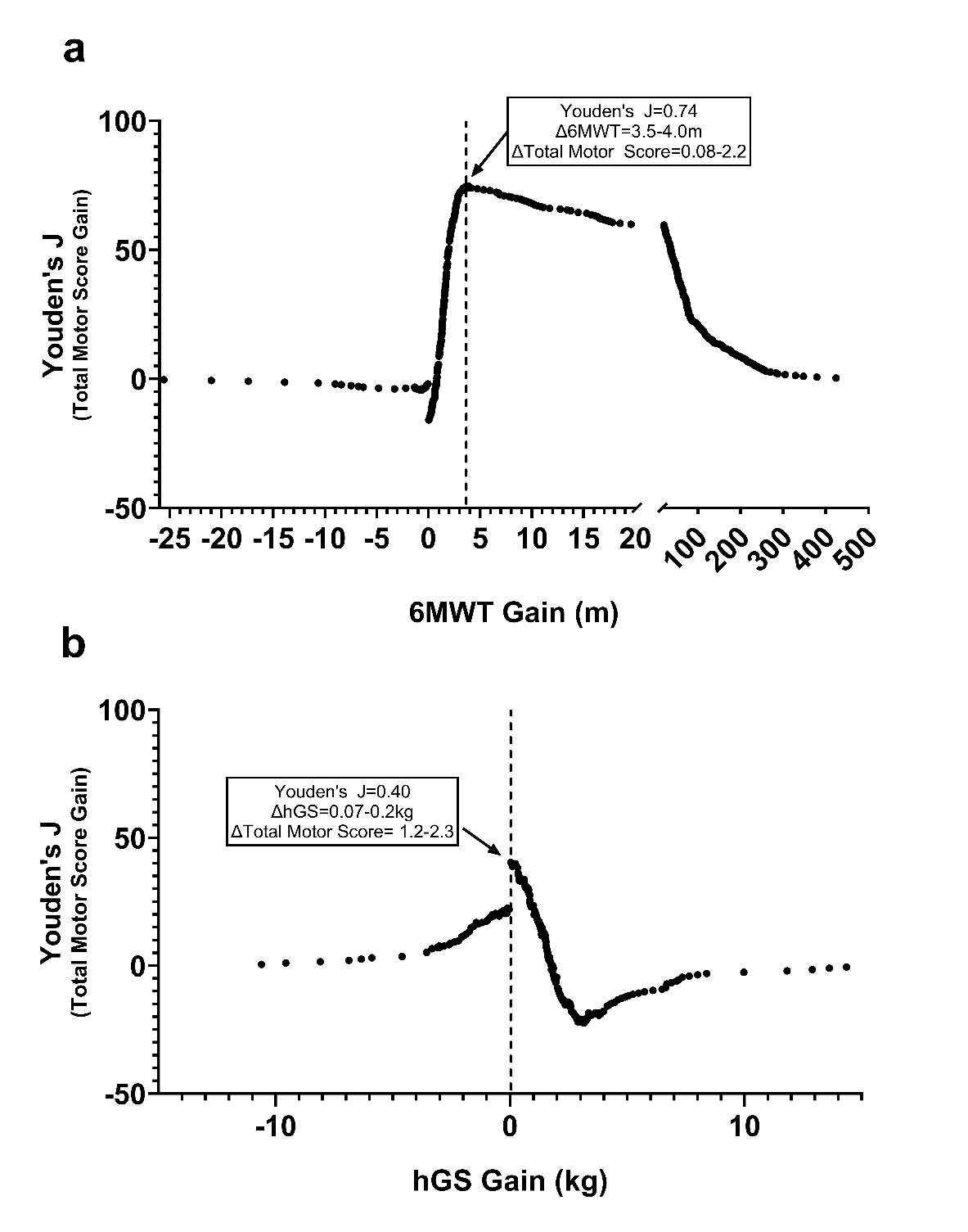
**

**Figure S1:** Youden’s J calculated from ROC-derived sensitivity and specificity values for a) 6MWT gain and b) hGS gain in their ability to predict Total Motor Score gain.

**Supplemental references**

1. S1. Loop, M.S., Lotspeich, S.C., Garcia, T.P., Meyer, M.L.. (2025). Should regression calibration or multiple imputation be used when calibrating different devices in a longitudinal study?. *American Journal of Epidemiology*, 194(1), 295–301.
